# Surgical Uses of Cocaine

**Published:** 1896-03

**Authors:** 


### Surgical Uses of Cocaine.

*Medical and Surgical Reporter* states that Dr. D. B. Merrill Ricketts has employed cocaine in an extensive variety of operations, and offers the following practical maxims as to its methods of application :

1. Solutions should not be made except at time of operation.

2. Cold water solutions will add materially to the good effects of the cocaine.

3. An operator should accustom himself to but one kind of cocaine, and confine himself to the use of that kind as he does chloroform.

4. The operator should prepare the solution himself, and not intrust it to an assistant.

5. Only small amounts should be used, especially in the young, the old, and in highly nervous persons.

6. Small quantities should be used in operations upon the face, head and neck.

7. Operations should be made as soon as possible after the area has become anæsthetized that as much as possible of the solution may escape through the wound, and thus not enter the general circulation.

8. The finest needle should be used, and its length should be sufficient to traverse the line of incision intra-cutaneously with but one puncture.

9. Intra-cutaneous and not sub-cutaneous injections should be made, because there is less of the drug absorbed, and because there is no pain to result from cutting tissue beneath the integument.

10. The nearer the injection is to the end of the member the less is the amount of absorption.

11. The prepuce, fingers, toes, hands and feet can be constricted in such a way as to prevent, to a considerable degree, the entrance of the solution into the general circulation.

